# *Bacidiaalbogranulosa* (Ramalinaceae, lichenized Ascomycota), a new sorediate lichen from European old-growth forests

**DOI:** 10.3897/mycokeys.44.30199

**Published:** 2018-12-14

**Authors:** Jiří M líček, Zdeněk alice, Jan Vondrák, Anna Łubek, Martin Kukwa

**Affiliations:** 1 Institute of Botany, The Czech Academy of Sciences, Zámek 1, CZ-252 43 Průhonice, Czech Republic; 2 Faculty of Sciences, Department of Botany, Charles University in Prague, Benátská 2, CZ-128 01 Praha 2, Czech Republic; 3 Faculty of Biological Sciences, University of South Bohemia, Branišovská 31, CZ-370 05 České Budějovice, Czech Republic; 4 Institute of Biology, Jan Kochanowski University in Kielce, Świętokrzyska 15A, PL-25-406 Kielce, Poland; 5 Faculty of Biology, Department of Plant Taxonomy and Nature Conservation, University of Gdańsk, Wita Stwosza 59, PL-80-308 Gdańsk, Poland

**Keywords:** Atranorin, sterile lichens, subneutral bark

## Abstract

A sterile sorediate member of the genus *Bacidia* s.str., *B.albogranulosa*, is described here as a new species. It is characterised by its very thin, pale grey thallus, white, farinose to granular soredia, the production of atranorin and the absence of ascomata and pycnidia. It grows on slightly acidic to subneutral bark of broad-leaved trees in old-growth forests in the Czech Republic, Poland, Ukraine and Russia (European part of the Caucasus). The new species is well characterised by its morphology, secondary chemistry and molecular (nrITS, mtSSU) traits. It is closely related to other atranorin-containing species in the genus, *Bacidiadiffracta*, *B.polychroa* and *B.suffusa*.

## Introduction

*Bacidia* De Not. (Ramalinaceae, lichenised Ascomycota) is a genus of lichenised fungi with crustose thalli, a chlorococcoid photobiont, lecideine or biatorine apothecia and multiseptate oblong to acicular ascospores (Ekman 1996). Many of the species do not produce any lichen substances detectable by TLC, but one or more pigments in the apothecial tissues are known (Ekman 1996, Coppins and Aptroot 2009, Wirth et al. 2013). Names of five acetone-insoluble pigments are derived from *Bacidia* s.str., i.e. Arceutina-yellow, Laurocerasi-brown, Polychroa-brown, Rubella-orange and Schweinitzii-red (Ekman 1996, Meyer and Printzen 2000). The genus *Bacidia* includes approximately 230 species wordwide (Lücking et al. 2016). However, many species named ‘*Bacidia*’ belong to other genera or even other families, so the diversity of *Bacidia*, in its strict sense, is estimated to be 60–90 species (Ekman 1996, 2001, Coppins and Aptroot 2009).

During field research in old-growth forests in Europe, we repeatedly collected a sterile sorediate crust, preliminarly assigned to the genus *Lecanora* Ach. due to the production of atranorin. Surprisingly, molecular data placed the species into *Bacidia* s.str. The currently known members of *Bacidia* s.str., except for *B.sorediata* Lendemer & R. C. Harris (Lendemer et al. 2016), do not produce isidia or soredia, but the thallus of some species consists of granules that very likely have a function as vegetative propagules (Ekman 1996). The new species is related to *B.diffracta* S. Ekman, *B.polychroa* (Th. Fr.) Körb., *B.rubella* (Hoffm.) A. Massal. and *B.suffusa* (Fr.) A. Schneid., which also produce atranorin as the main secondary metabolite (Culberson and Culberson 1969, Ekman 1996). Based on morphological, chemical and molecular characters, we describe this very distinct taxon as new to science.

## Material and methods

### Sampling, morphology and chemistry

Collected specimens are deposited in KTC, PRA, UGDA and the personal herbarium of J. Malíček. Microscopic descriptions are based on hand-cut sections mounted in water. Lichen secondary metabolites were identified using thin layer chromatography (TLC) in A, B’ and C solvents (Orange et al. 2010). Figures were acquired by the stereomicroscope Olympus SZX 12 with the cooled colour digital camera Olympus DP 70 (resolution 12.5 Mpx) in the software QuickPHOTO MICRO 3.0 (Promicra), using an extended depth of field module Deep Focus.

### DNA extraction, PCR amplification and sequencing

The Invisorb Spin Plant Mini Kit (Invitek) and CTAB protocol (Cubero et al. 1999) were used for DNA extractions. The fungal ITS rDNA (henceforth ITS) and mitochondrial SSU (mtSSU) were amplified with the following primers: ITS1F (Gardes and Bruns 1993) and ITS4 (White et al. 1990), mrSSU1, mr SSU2R and mrSSU3R (Zoller et al. 1999). PCR reactions of nrITS and mtSSU were prepared for a 20 µl final volume containing 14 µl double-distilled water, 4 µl MyTaq polymerase reaction buffer, 0.2 µl MyTaq DNA polymerase, 0.4 µl of each of the 25 mM primers and 1 µl of the sample. Amplifications of both loci consisted of an initial 1 min denaturation at 95 °C, followed by 35 cycles of 1 min at 95 °C, 1 min at 56 °C, 1 min at 72 °C and a final extension of 7 min at 72 °C. The PCR products were visualised on a 0.8% agarose gel and cleaned with GenElute PCR Clean-Up Kit (Sigma), according to the manufacturer’s protocols. In total, 5 new ITS and 8 mtSSU sequences were generated (Table 1). Two short mtSSU sequences, containing ca. 400 positions, were exluded from the final analysis.

**Table 1.** GenBank accession numbers and voucher information of specimens used in this study. New sequences are indicated in bold.

Taxon	Source – Specimen	ITS	mtSSU
*Bacidiaalbogranulosa* 1	Czech Republic, Lanžhot, J. Vondrák 11888 (PRA)	**MK158342**	**MK158332**
*Bacidiaalbogranulosa* 2	Czech Republic, Lanžhot, J. Vondrák 11889 (PRA)	**MK158341**	**MK158333**
*Bacidiaalbogranulosa* 3	Czech Republic, Šumava Mts, J. Vondrák 17113 (PRA)	**MK158339**	**MK158334**
*Bacidiaalbogranulosa* 4	Russia, Caucasus, J. Malíček 9622 (hb. J. Malíček)	**MK158340**	**MK158335**
*Bacidiaalbogranulosa* 5	Czech Republic, Moravský kras, J. Malíček 8013 (hb. J. Malíček)	−	**MK158336**
*Bacidiaalbogranulosa* 6	Ukraine, Otok, J. Vondrák 12235 (PRA)	−	**MK158337**
*Bacidiaalbogranulosa* 7	Czech Republic, Český les Mts, J. Vondrák 12865 (PRA)	−	**MK158338**
* Bacidia arceutina *	Switzerland, van den Boom 41117 (hb. van den Boom)	−	JQ796829
* Bacidia diffracta *	Wetmore 26401 (MIN)	AF282090	−
*Bacidiaekmaniana* 1	USA, Delaware, Lendemer 33783 (NY)	−	KX151745
*Bacidiaekmaniana* 2	USA, North Carolina, Lendemer 30488A (NY)	−	KX151746
* Bacidia fraxinea *	Sweden, Johansson 1620 (BG)	AF282088	−
* Bacidia polychroa *	Knutsson 91–215 (hb. Knutsson)	AF282089	−
* Bacidia rosella *	Sweden, Ekman 3117 (BG)	AF282086	AY300877
*Bacidiarubella* 1	Poland, Pojezierze Ilawskie, M. Kukwa 4598 (DUKE)	MG461695	DQ986808
*Bacidiarubella* 2	Ukraine, Otok, J. Vondrák 12200 (PRA)	**MK158343**	**MK158331**
*Bacidiarubella* 3	Switzerland, van den Boom 41103 (hb. van den Boom)	JQ796852	JQ796830
*Bacidiarubella* 4	Sweden, Ekman 3021 (BG)	AF282087	−
*Bacidiaschweinitzii* 1	USA, North Carolina, Lendemer 30548 (NY)	KX151761	KX151749
*Bacidiaschweinitzii* 2	USA, North Carolina, Tripp 2614 (NY)	KX151762	KX151750
*Bacidiaschweinitzii* 3	USA, North Carolina, Lendemer 29364 (NY)	KX151763	KX151751
*Bacidiaschweinitzii* 4	USA, North Carolina, Lendemer 31238 (NY)	KX151764	KX151752
*Bacidiaschweinitzii* 5	USA, Maryland, Lendemer 31855 (NY)	KX151765	KX151753
*Bacidiaschweinitzii* 6	USA, Tennessee, F. Lutzoni (DUKE)	DQ782850	DQ972998
* Bacidia sipmanii *	Tenerife, Sérusiaux s.n. (hb. Sérusiaux)	JQ796853	JQ796832
*Bacidiasorediata* 1	USA, Maryland, Lendemer 33869 (NY)	KX151773	KX151760
*Bacidiasorediata* 2	USA, North Carolina, Lendemer 35031 (NY)	KX151769	KX151756
*Bacidiasorediata* 3	USA, Delaware, Lendemer 33702 (NY)	KX151767	KX151754
*Bacidiasorediata* 4	USA, Delaware, Lendemer 33787 (NY)	KX151772	KX151759
*Bacidiasorediata* 5	USA, North Carolina, Lendemer 35386 (NY)	KX151770	KX151757
*Bacidiasorediata* 6	USA, Virginia, Lendemer 31692 (NY)	KX151768	KX151755
*Bacidiasorediata* 7	USA, Virginia, Lendemer 31527 (NY)	KX151771	KX151758
* Bacidia suffusa *	Wetmore 74771 (MIN)	AF282091	−
*Bacidinaarnoldiana* s.lat.	Poland, Pojezierze Ilawskie, M. Kukwa 4593 (DUKE)	HQ650650	DQ986810
* Toninia sedifolia *	Canada, Quebec, F. Lutzoni & J. Miadlikowska (DUKE)	HQ650689	DQ972987

### Sequence alignment and phylogenetic analysis

The newly produced sequences were edited in BioEdit 7.2.5 (Hall 1999). The final analyses included the newly generated sequences, the most similar *Bacidia* sequences (identity > 90%) according to a BLASTN search (Altschul et al. 1990) in the GenBank database and sequences of chemically and morphologically similar species (*B.schweinitzii* (Fr. ex Tuck.) A. Schneid., *B.sorediata*) to demonstrate their distant position in the tree. *Bacidinaarnoldiana* s.lat. and *Toniniasedifolia* (Scop.) Timdal were selected as an outgroup. The ITS and mtSSU regions were aligned separately using MAFFT 7 (Katoh and Standley 2013) with L-INS-i method (Katoh et al. 2005). Ambiguous positions were excluded from the analysis using Gblocks 0.91b (Castresana 2000), with a less stringent selection, on the Phylogeny.fr server (Dereeper et al. 2008). The final ITS alignment contained 443 positions and 29 sequences; the mtSSU alignment had 730 positions and 28 sequences. Gaps were coded in SeqState by simple coding (Simmons and Ochoterena 2000).

We concatenated the alignments and inferred a phylogeny using MrBayes 3.2.6 (Huelsenbeck and Ronquist 2001; Ronquist et al. 2012). Results of MrModeltest 2.0 (Nylander 2004) suggested the general time reversible model, including gamma-distributed rates across sites modelled with four discrete categories and a proportion of invariant sites (GTR+G+I), as the best substitution model for both regions. Each analysis was performed with two runs, each with four MCMC chains (temperature 0.05). Trees were sampled every 500^th^ generation. Analyses were stopped when the average standard deviation of the split frequencies between the simultaneous runs was below 0.01. To eliminate trees sampled before reaching apparent stationarity, the first 25% of entries were discarded as burn-in and the rest were used to compute a majority-rule consensus tree with Bayesian posterior probabilities for the branches.

A maximum likelihood analysis was performed using RAxML-HPC v. 8.2.10 (Stamatakis 2014) with the GTR+G+I model on the CIPRES Science Gateway (Miller et al. 2010). Non-parametric bootstrap analysis was performed with 1000 bootstrap replicates. The maximum likelihood consensus tree is not shown, but bootstrap values are indicated at branches in the Bayesian tree (Fig. 2).

## Results and discussion

### Taxonomy

#### Bacidia albogranulosa

Taxon classificationFungiLecanoralesRamalinaceae

Malíček, Palice, Vondrák & Kukwa sp. nov.

828612

Fig. 1

##### Type.

CZECH REPUBLIC. Dolnomoravský úval lowland: Břeclav, Lanžhot, protected area Cahnov, 150 m alt., 48°39'22"N, 16°56'25"E, on bark of *Acercampestre*, 1 Apr 2014, J.Vondrák (holotype: PRA-Vondrák 11888).

##### Diagnosis.

The species is characterised by a grey-white hypothallus or very thin thallus covered by groups of white, farinose to granular soredia or by being completely sorediate. Ascomata and pycnidia are unknown. Atranorin is the only secondary metabolite. The species occurs in old-growth forests on bark of broad-leaved trees with high bark pH (> 5).

##### Etymology.

The name refers to the white rough (granular) soredia that are often present.

**Figure 1.**
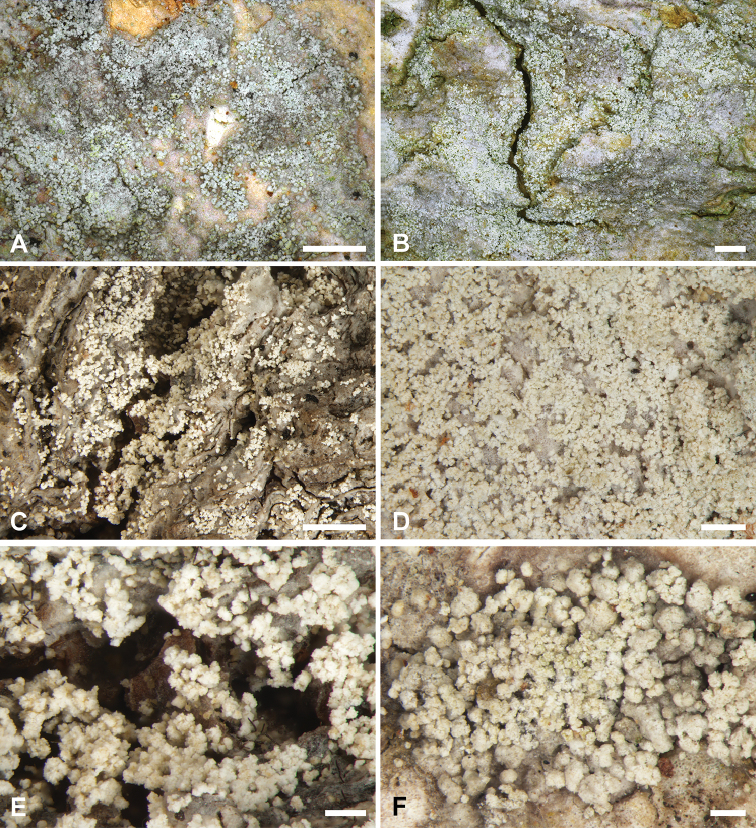
Morphology of *Bacidiaalbogranulosa*. **A** Holotype (PRA/Vondrák 11888) **B** Common phenotype (Malíček 10802) **C** Typical growth form on old beech trees (Malíček 8166) **D** Phenotype with abundant soredia forming a seemingly leprose thallus (Malíček 8013) **E** Detail of soredia (Malíček 8166) **F** Soredia arising from granules (PRA/Vondrák 11888). Scale bars: 1 mm (**A−C**), 0.5 mm (**D**), 0.2 mm (**E, F**). Photos by J. Malíček (**A, B**) and J. Machač (**C−F**).

##### Description.

The thallus consists of a hypothallus (i.e. without photobiont cells) or, in some parts, a lichenised and thinly episubstratal thallus (up to 100 μm high), which is smooth or partially areolate, pustulate or granular, grey-white to grey, sorediate. A prothallus is absent or very thin and white. Soredia are not produced in clearly delimited soralia, but dispersed in groups or forming a more or less continuous layer, white or, when fresh, yellowish-white, farinose to granular, simple, (25–)35–65 μm in diam., or in consoredia up to 125 μm in diam. Soredia are enclosed by a colourless, more or less compact “wall” without projecting hyphae. The photobiont is trebouxioid, and 5–16 μm in diameter. Ascomata and pycnidia are unknown.

##### Chemistry.

Atranorin detected by TLC (n=20). Numerous tiny crystals of atranorin visible in water mounts of soredia and thallus in polarised light. Spot reactions: K+ yellow, Pd+ yellowish, C–, KC–, soredia UV+ dull orange, thallus UV– or dull yellowish-white (in 365 nm).

##### Distribution and ecology.

The new species is reported from the Czech Republic, Poland, Russia (European part of the Caucasus) and Ukraine. It has already been published under a provisional name, *Bacidiaalbogranulosa* ined. from the Czech Republic (Vondrák et al. 2016) and the Ukrainian Carpathians (Malíček et al. 2018, Vondrák et al. 2018).

*Bacidiaalbogranulosa* grows abundantly in old-growth floodplain and scree forests in the Czech Republic and old-growth ash or hornbeam dominated broad-leaved forests in Poland. It rarely occurs in old-growth beech (Ukraine) and mixed forests (Russia). It has usually been found on a dry and coarse bark of broad-leaved trees with a relatively high bark pH (approximately > 5). The most frequent phorophytes are *Acercampestre* (n=5), *A.platanoides* (11) and *Fagusorientalis*/*sylvatica* (4; overmature or dying trees due to a fungal infection). A few specimens were recorded also on *Fraxinusangustifolia* (2), *F.excelsior* (2), *Carpinusorientalis* (1), *Euonymuseuropaeus* (1) and *Quercus* sp. (2). The species prefers rather shaded trunks and places not directly exposed to rain, similar to many *Lepraria* species (Saag et al. 2009).

*Alyxoriavaria* (Pers.) Ertz & Tehler, *Bacidiarubella* and the non-lichenised fungus *Dendrotheleacerina* (Pers.) P.A. Lemke (on *Acer* spp.) are the most commonly recorded, co-occurring species. In the Czech Republic, the new species was repeatedly found on weathered bark with the red-listed *Gyalectaflotowii* Körb. or *G.ulmi* (Sw.) Zahlbr. It co-occurred also with *Acrocordiagemmata* (Ach.) A. Massal., *Arthotheliumspectabile* A. Massal., *Bacidiafraxinea* Lönnr., *B.incompta* (Borrer) Anzi, *Caloplacaflavocitrina* (Nyl.) H. Olivier, *Gyalectatruncigena* (Ach.) Hepp, *Hazslinszkyagibberulosa* (Ach.) Körb., *Inodermabyssaceum* (Weigel) Gray, *Lecaniacroatica* (Zahlbr.) Kotlov, *Leprariafinkii* (B. de Lesd.) R. C. Harris, *L.vouauxii* (Hue) R. C. Harris, *Opegraphavermicellifera* (J. Kunze) J. R. Laundon and *Pyrenulanitidella* (Flörke ex Schaer.) Müll. Arg.

##### Phylogeny.

**Figure 2.**
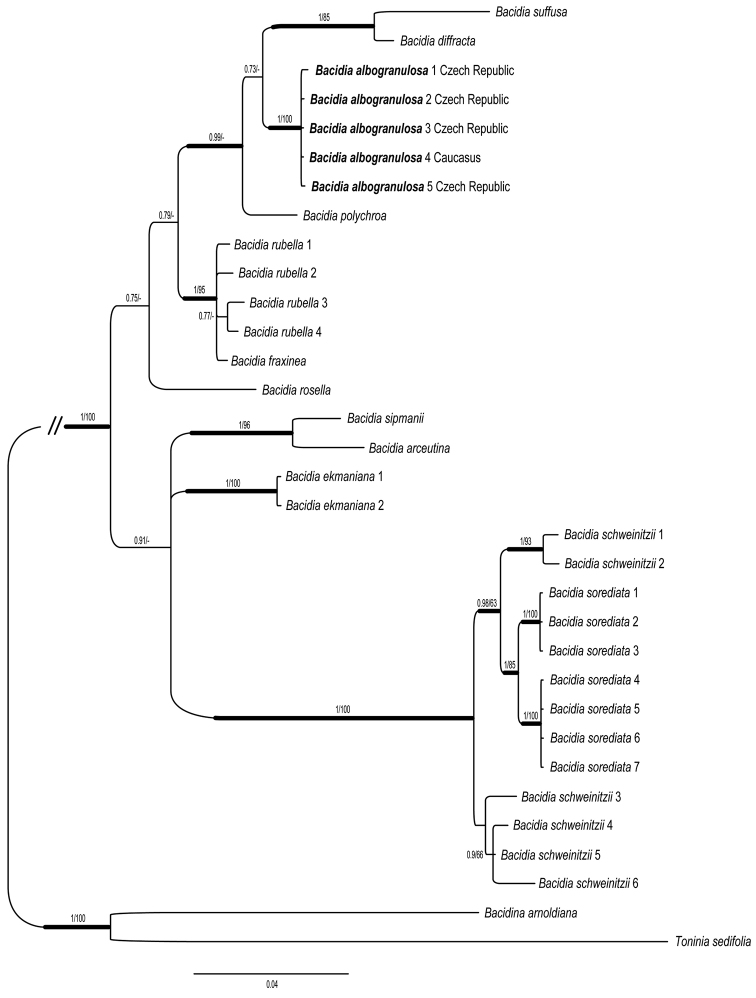
Phylogeny of selected members of *Bacidia* s.str. This is a Bayesian phylogenetic reconstruction based on nrITS and mtSSU sequences. The new species, *Bacidiaalbogranulosa*, is indicated in bold. Branches with > 0.95 Bayesian posterior probability values are indicated by thicker lines. Bayesian posterior probabilities (first value) and maximum likelihood bootstrap percentages (second value) are indicated.

The new species is strongly supported as a distinct clade in the ITS and mtSSU phylogeny (Fig. 2) and belongs to *Bacidia* s.str. sensu Ekman (2001). According to the ITS data, it is closely related to *Bacidiadiffracta*, *B.suffusa* and *B.polychroa*. These four species form a well supported group, characterised by the presence of the pigments Laurocerasi-brown and Polychroa-brown in the apothecia. *Bacidiaalbogranulosa* is also related to *B.rubella*, a species it frequently co-ocurrs with. The only sorediate member of *Bacidia* s.str., the North American *B.sorediata*, seems not to be closely related to the new species, based on the ITS and mtSSU sequence data (Fig. 2).

##### Notes.

Although apothecia and pycnidia are unknown, *B.albogranulosa* can be recognised in the field by its white-grey hypothallus or very thin thallus covered by groups of white to yellowish-white soredia that often extend across the entire thallus. Ecologically, the species prefers trees with rough and slightly acidic or subneutral bark in old-growth forests.

*Bacidiaalbogranulosa* may macroscopically resemble some *Lepraria* species or poorly developed *Phlyctisargena* (Ach.) Flot., but it clearly differs by having a non-continuous, locally developed thallus, composed of dispersed granular aggregates that disintegrate into soralia at an early stage and by the lack of a fibrous prothallus. Additionally, atranorin alone is not known from any described *Lepraria* species (Saag et al. 2009). Similarly, sorediate European *Lecanora* species contain other substances in addition to atranorin, such as aliphatic acids, depsides/depsidones or terpenoids and usually form thicker thalli or at least a distinct fibrous hypothallus (Malíček et al. 2017). A slightly similar appearance is typical for a few other *Lecanora* species (e.g. *L.compallens* Herk & Aptroot, *L.stanislai* Guzow-Krzem., Łubek, Malíček & Kukwa), producing usnic acid and zeorin and forming a yellowish-greenish to greenish-grey sorediate thallus (Guzow-Krzemińska et al. 2017).

Initial stages of the new species may resemble sterile thalli of *Caloplacasubsterilis* Vondrák, Palice & van den Boom. This taxon lacks atranorin and tends to form thin areolate-squamulose, almost evanescent thalli with occassional sulcate or marginal soralia (Vondrák et al. 2013). The closely related species *B.diffracta* produces a similar, finely granular grey thallus and contains atranorin in addition to traces of zeorin. Nevertheless, this species is richly fertile, has larger thalline granules (40–100 μm diam.) and is so far only known from eastern North America (Ekman 1996). The only presently known sorediate member of *Bacidia* s.str., *B.sorediata*, differs in having a better developed, grey-green to dark green thallus, diffuse, rarely confluent soralia and fine soredia. It occurs only in south-eastern North America (Lendemer et al. 2016) and it is not phylogenetically closely related to *B.albogranulosa* (Fig. 2).

##### Additional specimens examined.

CZECH REPUBLIC. Western Bohemia: Český les Mts, Bělá nad Radbůzou, Smolov, protected area Pleš, old-growth mixed forest on scree on E slope, 49°33'02"N, 12°38'21"E, 740–840 m alt., on *Acerplatanoides*, 6 August 2014, J.Vondrák 12865 (PRA). Southern Bohemia: Šumava Mts, Volary, Mt Stožec – Medvědice, a mountain scree deciduous old-growth forest at NNE-facing slope, 48°52'49.5"N, 13°50'03"E, on dry bark of *Acerplatanoides*, 935 m alt., 7 Aug 2014, Z.Palice 17827, Jul.Palicová & K.Palicová (PRA), ibid.: at NE-facing slope, 48.8802°N, 13.8385°E, 900 m alt., on bark of *Acerplatanoides*, 17 Oct 2016, J.Vondrák 17113 & Z.Palice 24362 (PRA). Šumava Mts, Lenora, Mt Zátoňská hora, semi-natural scree deciduous forest at SW-facing slope, just below the top, 48°56'41"N, 13°49'48"E, on bark of *Acerplatanoides*, 1022 m alt., 27 June 2018, J.Malíček & Z.Palice 25133 (PRA). Novohradské hory Mts, Pohorská Ves, nature reserve Žofínský prales, N part of the reserve, old-growth forest at N-NW-facing slope, 48°40'10"N, 14°42'30"E, on bark of *Fagus*, 765–770 m alt., 18 Aug 2016, Z.Palice 22220 (PRA). Central Bohemia: Křivoklátsko Protected Landscape Area, Skryje, Týřov National Nature Reserve, mixed deciduous forest with shady rocky outcrops in valley of Úpořský potok brook S of Vápenný vrch Hill (424 m), 49°58'09"N, 13°47'43"E, 270 m alt., on bark of *Acerplatanoides*, 11 Aug 2018, J.Malíček 11990 (herb. Malíček). Southern Moravia: distr. Břeclav, Lanžhot, Ranšpurk National Nature Reserve, ca. 48°40'41"N, 16°56'49"E, floodplain old-growth forest, alt. 150 m, on bark of *Acercampestre*, 10 Oct 2013, J.Malíček 6214 & J.Vondrák (herb. Malíček). Cahnov-Soutok National Nature Reserve, old-growth flood-plained forest 7.5 km SSW of Lanžhot, 48°39'23"N, 16°56'24"E, 150 m alt., on bark of *Acercampestre* and *Fraxinusangustifolia*, 1–3 Apr 2014, J.Malíček 6793, 6832, 6863, M.Kukwa 12409, 12434, 12504, 12514, 12515, 12526, Z.Palice 17686 & J.Vondrák 11889, 12051, 12057, (herb. Malíček, PRA, UGDA). Distr. Blansko, Moravský kras Protect. Landscape Area, Vilémovice, Vývěry Punkvy National Nature Reserve, oak-dominated woodlands on SE-facing slope in surrounding of Blansek castle ruin, 49°22'15"N, 16°43'24"E, alt. 425 m, on bark of *Acercampestre*, 17 Apr 2015, J.Malíček 8013 & V.Lenzová (herb. Malíček).

POLAND. Równina Bielska: Białowieża Primeval Forest, Białowieski National Park, N part of forest section no 286, 52°45'07"N, 23°52'40"E, *Tilio*-*Carpinetum*, on *Acerplatanoides*, May 2014, M.Kukwa 12592 (UGDA); ibid.: forest section no 256, *Tilio*-*Carpinetum*, on *Acerplatanoides*, May 2014, M.Kukwa 12755 (UGDA); ibid.: *Circaeo*-*Alnetum*, on *Acerplatanoides* and bark of fallen *Fraxinusexcelsior*, Aug 2014, M.Kukwa 13135a, 13176 & A.Łubek (KTC, UGDA); ibid.: *Tilio*-*Carpinetum*, on *Acerplatanoides*, August and October 2014, M.Kukwa 13292, 14394 & A.Łubek (KTC, UGDA); ibid.: *Tilio*-*Carpinetum*, on *Acerplatanoides*, Aug 2015, M.Kukwa 17195, 17584, 17404 & A.Łubek (KTC, UGDA); ibid.: *Circaeo*-*Alnetum*, on bark of log (*Fraxinusexcelsior*), 24 Aug 2015, M.Kukwa 17446 & A. Łubek (KTC, UGDA).

RUSSIA. Caucasus Mts: Caucasian Biosphere Reserve, Guzeripl’, old-growth deciduous mixed forest (*Quercusrobur*, *Alnusglutinosa*, *Acercampestre* etc.) at right bank of Belaya River, 0.4 km WSW of margin of village, 43°59'20"N, 40°07'30"E, 700 m alt., on bark of *Acercampestre*, *Carpinusorientalis*, *Fraxinus* and *Quercusrobur*, 8–9 June 2016, J.Malíček 9622, 10491, Z.Palice 21600, 21690, 22395, 22622, 22715, 23063, J.Vondrák 14956 & G.Urbanavichus (herb. Malíček, PRA). Guzeripl’, a forested crest between Belaya and Molchepa rivers, just ca. 1 km SSE of the village, well-lit mixed forest at N-wards descending crest, 43°59'12"N, 40°08'30"E, on bark of old *Quercus*, 935 m alt., 7 June 2016, Z.Palice 22672, 22964 & J.Vondrák 15532 (PRA). Guzeripl’, mixed primeval forest (*Abiesnordmanniana*, *Acertrautvetteri*, *Fagusorientalis* etc.) on a ridge and W-facing slope 3.5 km S of village, 43°57'53"N, 40°07'50"E, 1470 m alt., on bark of *Acerplatanoides* and *Fagusorientalis*, 14 June 2016, J.Malíček 10802, Z.Palice 22624, 22924, J.Vondrák 15291 & G.Urbanavichus (herb. Malíček, PRA).

UKRAINE. Zakarpattia Oblast Province: Berehovo, Nyzhni Remety: lood-plain forest “Otok” 2.5 km SW of village, close to Mala Borzhava River, 48°14'12"N, 22°48'25"E, 120 m alt., on bark of *Acercampestre*, 23 Oct 2013, J.Malíček 6463 & J.Vondrák (herb. Malíček). Ibid.: “Otok”, ca. 4 km SW of village, 48°14'00"N, 22°48'20"E, 190 m alt., on bark of *Acercampestre*, *Euonymuseuropaeus* and *Fraxinusangustifolia*, 3 June 2014, J.Šoun & J.Vondrák 12235, 12206, 12237 (PRA). Khust, Velyka Uhol’ka, old-growth beech predominated forest in valley of Velika Uhol’ka River, ca. 0.7 km NNE of last houses in village, 48°15'02"N, 23°41'47"E, 500 m alt., on bark of old *Fagussylvatica*, 13 May 2015, J.Malíček 8166 & Z.Palice 19366 (herb. Malíček, PRA); ibid.: old-growth hornbeam-beech forest, 48°14'43"N, 23°41'39"E, on bark of old *Fagussylvatica*, 460 m alt., 19 May 2015, Z.Palice 19392 (PRA).

## Supplementary Material

XML Treatment for Bacidia albogranulosa
